# Risk of Adverse Events in Anticoagulated Patients With Atrial Fibrillation and Nonalcoholic Fatty Liver Disease

**DOI:** 10.1210/clinem/dgae394

**Published:** 2024-06-12

**Authors:** Tommaso Bucci, Katarzyna Nabrdalik, Francesco Baratta, Daniele Pastori, Pasquale Pignatelli, Theresa Hydes, Uazman Alam, Francesco Violi, Gregory Y H Lip

**Affiliations:** Liverpool Centre for Cardiovascular Science at University of Liverpool, Liverpool John Moores University and Liverpool and Heart and Chest Hospital, Liverpool L7 8TX, UK; Department of Clinical Internal, Anesthesiological and Cardiovascular Sciences, Sapienza University of Rome, Rome 00185, Italy; Liverpool Centre for Cardiovascular Science at University of Liverpool, Liverpool John Moores University and Liverpool and Heart and Chest Hospital, Liverpool L7 8TX, UK; Department of Internal Medicine, Diabetology and Nephrology, Faculty of Medical Sciences in Zabrze, Medical University of Silesia, Katowice 40-055, Poland; Department of Clinical Internal, Anesthesiological and Cardiovascular Sciences, Sapienza University of Rome, Rome 00185, Italy; Department of Clinical Internal, Anesthesiological and Cardiovascular Sciences, Sapienza University of Rome, Rome 00185, Italy; Department of Clinical Internal, Anesthesiological and Cardiovascular Sciences, Sapienza University of Rome, Rome 00185, Italy; Liverpool Centre for Cardiovascular Science at University of Liverpool, Liverpool John Moores University and Liverpool and Heart and Chest Hospital, Liverpool L7 8TX, UK; Diabetes & Endocrinology Research and Pain Research Institute, Institute of Life Course and Medical Sciences, University of Liverpool and Liverpool University Hospital NHS Foundation Trust, Liverpool L69 7ZX, UK; Liverpool Centre for Cardiovascular Science at University of Liverpool, Liverpool John Moores University and Liverpool and Heart and Chest Hospital, Liverpool L7 8TX, UK; Diabetes & Endocrinology Research and Pain Research Institute, Institute of Life Course and Medical Sciences, University of Liverpool and Liverpool University Hospital NHS Foundation Trust, Liverpool L69 7ZX, UK; Department of Clinical Internal, Anesthesiological and Cardiovascular Sciences, Sapienza University of Rome, Rome 00185, Italy; Liverpool Centre for Cardiovascular Science at University of Liverpool, Liverpool John Moores University and Liverpool and Heart and Chest Hospital, Liverpool L7 8TX, UK; Danish Center for Health Services Research, Department of Clinical Medicine, Aalborg University, Aalborg 9000, Denmark

**Keywords:** atrial fibrillation, non-alcoholic fatty liver disease, cardiovascular events, bleeding, all-cause death

## Abstract

**Background:**

The clinical impact of nonalcoholic fatty liver disease (NAFLD) in patients with atrial fibrillation (AF) is still controversial.

**Aim:**

To evaluate the 1-year risk of all-cause death, thromboembolic events, and bleeding in patients with AF-NAFLD.

**Methods:**

Retrospective study with a health research network (TriNetX). Patients with AF on oral anticoagulation (OAC) were categorized according to the presence of NAFLD into 2 groups. The primary outcomes were the 1-year risks of (1) a composite cardiovascular outcome (all-cause death, myocardial infarction, stroke, cardiac arrest, and pulmonary embolism) and (2) a composite hemorrhagic outcome (intracranial hemorrhage and gastrointestinal bleeding). Cox regression analysis before and after propensity score matching was used to estimate hazard ratio (HR) and 95% 95% CI,. Sensitivity analyses investigated the risk associated with cirrhosis, thrombocytopenia, and type of OAC (warfarin vs non-vitamin K antagonist oral anticoagulants (NOACs).

**Results:**

We identified 22 636 patients with AF-NAFLD (69 ± 12 years, 46.7% females) and 391 014 patients with AF and without liver disease (72 ± 12 years, 42.7% females). NAFLD was associated with a higher risk of composite cardiovascular (HR, 1.54; 95% CI, 1.47-1.61) and hemorrhagic (HR, 1.56; 95% CI, 1.42-1.72) outcomes. This was consistent also for all the single outcomes. Cirrhotic and thrombocytopenic patients with AF-NAFLD showed the highest risks. Compared to patients with AF-NAFLD on NOACs, those on warfarin were associated with a higher risk of cardiovascular and hemorrhagic outcomes.

**Conclusion:**

In patients with AF, NAFLD is associated with a higher 1-year risk of adverse events, with the risk of adverse events progressively increasing from noncirrhotic to cirrhotic and from nonthrombocytopenic to thrombocytopenic patients. NOACs were associated with a better effectiveness and safety profile compared to warfarin.

Atrial fibrillation (AF) is the most common cardiac arrhythmia worldwide and is associated with a significative increased risk of death and thromboembolic events (1). Oral anticoagulation (OAC), with vitamin K antagonists or non-vitamin K anticoagulants (NOACs), has been associated with a reduced risk of thrombotic events and death in patients with AF, but concerns have been raised in clinical contexts characterized by an increased hemorrhagic risk (2-5).

Both the risks of thromboembolic and hemorrhagic events in patients with AF on OAC are associated with the presence of other comorbidities, including the components of the metabolic syndrome (ie, diabetes, hypertension, dyslipidemia, and obesity) (6, 7). The latter metabolic risk factors beyond their role in increasing the risk of adverse events in patients with AF have been associated with a proinflammatory state characterized by insulin resistance and triglycerides accumulation in hepatocytes that configure the so called nonalcoholic fatty liver disease (NAFLD) (8). In Western countries, NAFLD represents the leading cause of liver disease and has been associated with a high risk of cardiovascular events and liver-related adverse events (9). However, no established strategies to address cardiovascular risk in NAFLD are available (10). Moreover, preclinical evidence and experimental models showed that NAFLD could affect liver-mediated drug metabolism raising possible concerns about OAC management (11, 12).

The aim of this study was to assess the 1-year risk of cardiovascular events and bleeding in patients with AF-NAFLD treated by OAC, using a global federated dataset.

## Methods

TriNetx is a research network used for several scientific purposes, compliant with the Health Insurance Portability and Accountability Act and the US federal law that protects the privacy and security of health care data, including deidentified data per the deidentification standard of the Health Insurance Portability and Accountability Act Privacy Rule (https://trinetx.com/real-world-resources/publications/). To gain access to the data in the TriNetX research network, requests are directed to TriNetX and a data sharing agreement is required. As a federated research network, studies using the TriNetX health research network do not need ethical approval because no patient identifiable identification is received. Further information about the data extraction from TriNetX is reported in the supplementary material.

### Study Design

This was a retrospective observational study conducted within TriNetX, a global federated health research network with access to electronic medical records from participating health care organizations including academic medical centers, specialty physician practices, and community hospitals covering approximately 80 million individuals, mainly in the United States. Within this network, available data include demographics, diagnoses using International Classification of Diseases, Tenth Revision, Clinical Modification (ICD-10-CM) codes, measurements (coded to Logical Observation Identifiers Names and Codes), and medications (Anatomical Therapeutic Chemical code). More information can be found online (https://trinetx.com/company-overview/).

### Cohort

The searches on the TriNetX online research platform were performed on October 5, 20235 for individuals aged ≥18 years with AF or flutter (ICD-10-CM code I48) and NAFLD (ICD-10-CM codes K75.81: Nonalcoholic steatohepatitis; K76.0: Fatty (change of) liver, not elsewhere classified; K76.89: Other specified diseases of liver) on oral anticoagulation (warfarin, edoxaban, rivaroxaban, apixaban, dabigatran). Exclusion criteria were history of alcohol abuse or alcoholic liver disease, chronic viral and autoimmune hepatitis, and biliary cirrhosis. More information about the ICD-10-CM codes used for the inclusion and exclusion criteria can be found in Supplementary Table S1 (13). To include the highest number of patients possible, the searches were not restricted to a specific period; however, more than 95% of patients considered in this study were entered in the TriNetX platform between 2000 and 2022. At the time of the search, 80 participating health care organizations had data available for patients who met the study inclusion criteria. The baseline index event date was the AF diagnosis; any other diagnoses or treatment reported on this date were the individual's baseline characteristics.

### Outcomes

The primary outcomes were the 1-year risk of a composite (1) cardiovascular outcome of all-cause death, acute myocardial infarction, ischemic stroke, peripheral arterial embolism, cardiac arrest, deep vein thrombosis of lower extremities, and pulmonary embolism, and (2) hemorrhagic outcome of intracranial hemorrhage (ICH) and gastrointestinal bleeding. The secondary outcomes were the 1-year risk of each component of the primary outcomes. The adverse events of interest were identified via ICD-10-CM codes (Supplementary Table S2 (13)).

In anticoagulated patients with AF, liver diseases and thrombotic and hemorrhagic events can be influenced by several factors. For this reason, we have performed different sensitivity analyses to test the reproducibility of the results obtained by the main analysis.

In the first sensitivity analyses, we evaluated separately the risk of primary outcomes in patients with AF-NAFLD based on sex, compared to patients with AF without liver diseases. Then, we directly compared male patients with AF-NAFLD with female patients with AF-NAFLD.

In the second sensitivity analyses, we evaluated separately the risk of primary outcomes in patients with AF-NAFLD with and without obesity (ICD-10-CM E66), compared to patients with AF without liver diseases. Then, we directly compared AF-NAFLD with obesity with those without obesity.

In the third sensitivity analyses, we evaluated separately the risk of primary outcomes in patients with AF-NAFLD with and without cirrhosis, compared to patients with AF without liver diseases. Then, we directly compared AF-NAFLD with cirrhosis with those without cirrhosis.

In the fourth sensitivity analysis, we analyzed separately the risk of primary outcomes in patients with AF-NAFLD with and without thrombocytopenia, compared to patients with AF without liver diseases. Then, we directly compared AF-NAFLD with thrombocytopenia with those without thrombocytopenia.

In the fifth sensitivity analysis, we compared patients with AF-NAFLD with patients with AF without liver diseases, considering separately those treated with warfarin and NOAC. Thereafter, we directly compared patients with AF-NAFLD on warfarin with those on NOAC.

### Statistical Analysis

All statistical analyses were performed on the TriNetX online research platform. Baseline characteristics were compared using chi-square tests for categorical variables and independent-sample *t*-tests for continuous variables. To create balanced cohorts, we performed a propensity score matching analysis (PSM), using logistic regression with a 1:1 greedy nearest neighbor matching model. Any baseline characteristic with an absolute standardized mean difference between cohorts lower than 0.1 was considered well matched. We included the following variables in the PSM: age, sex, ethnicity, arterial hypertension, ischemic heart diseases, ischemic stroke, heart failure, pulmonary hypertension, diabetes, and cardiovascular medications (including β-blockers, antiarrhythmics, diuretics, antilipemic agents, antianginals, calcium channel blockers, angiotensin-converting enzyme inhibitors, angiotensin II receptors blockers, and antiplatelets). Cox proportional hazard models was used to calculate hazard ratios (HRs) and 95% CI for the risk of adverse events in patients with AF-NAFLD before and after PSM.

Moreover, a competitive risk analysis was conducted using the Aalen–Johansen plot to determine the 1-year cumulative incidence of all-cause death, cardiovascular events, and bleeding events separately among patients with AF with and without NAFLD before PSM. The probability of death posing a significant competitive risk compared to another outcome was deemed high if, by the end of the follow-up period, death exhibited a greater cumulative incidence than the other outcome, or if there was an intersection between the cumulative incidence curves for death and the other outcome.

All tests were 2-tailed and *P* values of <.05 were taken to indicate statistical significance. All analyses were performed in the TriNetX platform which uses R's survival package v3.2-3.

## Results

The initial cohorts consisted of 22 636 patients with AF-NAFLD (mean age, 68.9 ± 12.2 years; females, 46.7%) and 391 014 patients with AF without liver diseases (mean age, 71.7 ± 11.9 years; females, 42.7%). The baseline comparisons between patients with AF-NAFLD with patients with AF without liver disease are reported in Table 1. Patients with AF-NAFLD were younger and showed a higher prevalence of obesity, diabetes, dyslipidemia, and chronic kidney disease, yet were less likely to have received prescriptions for antihypertensive, antilipemic, and antiplatelet drugs compared to patients with AF without liver diseases. The number of outcomes reported before PSM in patients with AF-NAFLD and in those without liver disease is shown in Table 2.

**Table 1.** Baseline clinical characteristics of patients with atrial fibrillation and nonalcoholic fatty liver disease compared to those with atrial fibrillation without liver diseases before and after propensity score matching

	Before propensity score matching			After propensity score matching		
	AF patients with NAFLD n = 22 636	AF patients without liver disease n = 391 014	ASD	AF patients with NAFLD n = 13 250	AF patients without liver disease n = 13 250	ASD
Age, y (±SD)	68.9 ± 12.2	71.7 ± 11.9	0.227	69.3 ± 12.0	69.2 ± 12.3	0.008
Female, n (%)	10 527 (46.7)	164 736 (42.7%)	0.081	5964 (45.0)	6018 (45.4)	0.008
White	17 505 (77.6)	295 674 (76.6)	0.024	10 163 (76.7)	10 181 (76.8)	0.003
Black or African American	2091 (9.3)	28 140 (7.3%)	0.072	1096 (8.3)	1113 (8.4)	0.005
Hypertension	13 374 (59.3)	210 359 (59.3)	0.097	8257 (62.3)	8250 (62.3)	0.001
Obesity	4087 (18.1)	38 621 (10.0)	0.235	2502 (18.9)	2358 (17.8)	0.028
Diabetes mellitus	6856 (30.4)	78 607 (20.4)	0.232	4074 (30.7)	4154 (31.4)	0.013
Dyslipidemia	8844 (39.2)	132 786 (34.4)	0.100	5511 (41.6)	5564 (42.0)	0.008
Chronic kidney disease	4171 (18.5)	44 040 (11.4)	0.200	2459 (18.6)	2333 (17.6)	0.025
Pulmonary hypertension	2589 (11.5)	22 935 (5.9)	0.197	1558 (11.8)	1421 (10.7)	0.033
Ischemic heart disease	6524 (28.9)	97 542 (25.3)	0.082	3994 (30.1)	8866 (29.2)	0.021
Heart failure	5827 (25.8)	85 074 (22.0)	0.089	3597 (27.1)	3309 (25.0)	0.050
Cerebrovascular diseases	971 (4.3)	16 223 (4.2)	0.039	605 (4.6)	621 (4.7)	0.006
Beta blockers	7635 (33.9)	214 517 (55.6)	0.448	6497 (49.0)	6543 (49.4)	0.007
Diuretics	4922 (21.8)	134 226 (34.8)	0.290	4411 (33.3)	4309 (32.5)	0.037
Calcium channel blockers	3835 (17.0)	105 244 (27.3)	0.249	3287 (24.8)	3293 (24.9)	0.001
ACE inhibitors	1866 (8.3)	66 664 (17.3)	0.272	1641 (12.4)	1573 (11.9)	0.016
Angiotensin II receptor blockers	1710 (7.6)	52 904 (13.7)	0.200	1479 (11.2)	1477 (11.1)	<0.001
Antiplatelet drugs	3839 (17.0)	97 801 (25.3)	0.205	3243 (24.5)	3163 (23.9)	0.014

Abbreviations: ACE, angiotensin-converting enzyme; AF, atrial fibrillation; ASD, absolute standardized mean difference; NAFLD, nonalcoholic fatty liver disease.

**Table 2.** 1-year risk of adverse events in patients with atrial fibrillation and nonalcoholic fatty liver disease compared to those with atrial fibrillation without liver diseases before and after propensity score matching

	Before propensity score matching			After propensity score matching		
	AF NAFLD n = 22 636	AF without liver disease n = 391 014	HR (95% CI)	AF NAFLD n = 13 250	AF without liver disease n = 13 250	HR (95% CI)
	Eventsn (%)	eventsn (%)		events n (%)	events n (%)	
**Composite cardiovascular outcome**	7900 (34.9)	67 658 (17.4)	2.17 (2.12-2.22)	4427 (33.4)	3046 (23.0)	1.54 (1.47-1.61)
All-cause death	3255 (14.4)	28 771 (7.4)	1.93 (1.86-2.00)	1922 (14.5)	1127 (8.5)	1.74 (1.62-1.87)
Stroke and peripheral embolism	1696 (7.5)	19 782 (5.1)	1.45 (1.38-1.53)	993 (7.5)	895 (6.8)	1.12 (1.02-1.22)
Myocardial infarction	1681 (7.4)	15 156 (3.9)	1.90 (1.80-1.99)	981 (7.4)	697 (5.3)	1.43 (1.30-1.58)
Cardiac arrest	461 (2.0)	3656 (0.9)	2.12 (1.93-2.34)	274 (2.1)	169 (1.3)	1.64 (1.35-1.98)
Deep vein thrombosis with pulmonary embolism	3333 (14.7)	14 919 (3.8)	3.97 (3.82-4.122)	1,74 (13.1)	980 (7.4)	1.83 (1.69-1.98)
**Composite hemorrhagic outcome**	1840 (8.1)	14 734 (2.8)	2.15 (2.05-2.56)	1010 (7.6)	664 (5.0)	1.56 (1.42-1.72)
Intracranial hemorrhage	370 (1.6)	4056 (1.0)	1.56 (1.38-1.71)	197 (1.5)	150 (1.1)	1.33 (1.07-1.64)
Gastro-intestinal bleeding	1511 (6.7)	10 932 (2.8)	2.37 (2.25-2.51)	835 (6.3)	528 (4.0)	1.62 (1.45-1.81)

Abbreviations: AF, atrial fibrillation; HR, hazard ratio; NAFLD, nonalcoholic fatty liver disease; PSM, propensity score matching.

**Figure 1.**
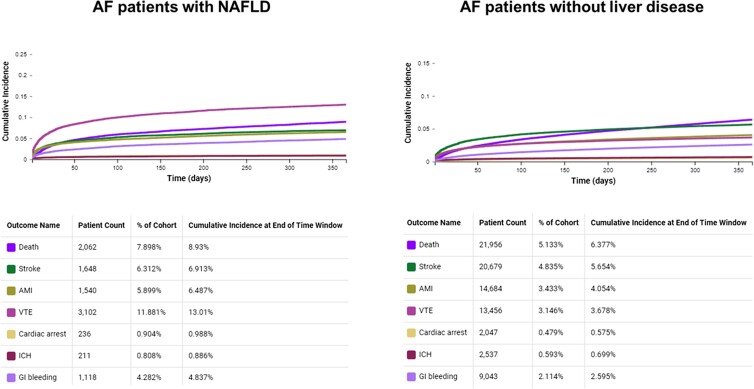
1-year Aalen–Johansen cumulative incidence curves for all-cause death and cardiovascular and hemorrhagic outcomes.

Patients with AF and NAFLD were at higher risk for both the composite cardiovascular (HR, 2.17; 95% CI, 2.12-2.22) and hemorrhagic (HR, 2.15; 95% CI, 2.05-2.56) outcomes. Regarding the secondary outcomes, patients with AF-NAFLD were associated with a higher risk of all-cause death, stroke, peripheral arterial embolism, myocardial infarction, cardiac arrest, deep venous thrombosis and/or pulmonary embolism, intracranial hemorrhages, and gastrointestinal bleeding compared to patients with AF without liver disease (Table 2). Analyzing the Aalen–Johansen plot, we found that the 1-year cumulative incidence of all-cause death was 8.9% in patients with AF and NAFLD and 6.4% in patients with AF without liver disease (Fig. 1).

In patients with AF and NAFLD, almost all bleeding events and cardiovascular events, except deep venous thrombosis and/or pulmonary embolism, showed a lower incidence compared to all-cause death. An evident over crossing within the first 50 days of follow-up was detectable for stroke and myocardial infarction (Fig. 1).

In patients with AF without liver disease, all the cardiovascular and hemorrhagic events had a lower 1-year cumulative incidence compared to that for all-cause death. There was a crossing point between the incidence projection of all-cause death and stroke showed after 250 days of follow-up (Fig. 1).

### Comparisons After Propensity Score Matching

**Figure 2.**
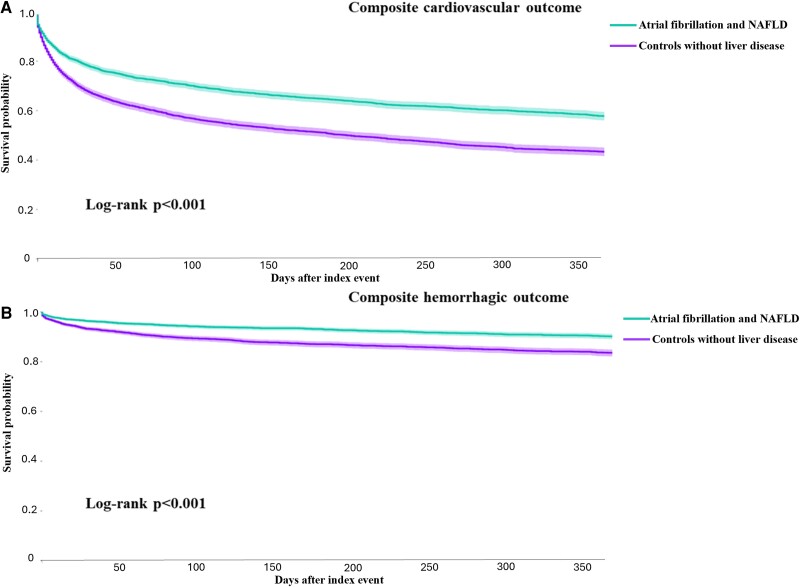
Survival curves for the primary outcomes in patients with atrial fibrillation and non-alcoholic fatty liver disease and controls patients without liver disease.

After PSM on a 1:1 ratio, 13 250 patients were selected for each group and no significant differences were found between these groups (Table 1). The number of adverse events after 1 year of follow-up is reported in Table 2. Compared to patients with AF without liver disease, patients with AF-NALFD were found to have a higher risk of composite cardiovascular (HR, 1.54; 95% CI, 1.47-1.61; Figure 2A) and hemorrhagic (HR, 1.56; 95% CI, 1.42-1.72; Figure 2B) outcomes, as well as the risks for each secondary outcome, consistent with the unmatched analysis (Table 2).

### Sensitivity Analyses

In the first sensitivity analysis, after PSM were selected for each group: 8010 for the comparison between patients with AF-NAFLD and those with AF without liver disease only in males; 6943 for the comparison between patients with AF-NAFLD and patients with AF without liver disease only in males; and 10 752 for the comparison between male patients with AF-NAFLD and female patients with AF-NAFLD (Supplementary Tables S3-S5 (13)). The number of events reported after 1 year of follow-up in all the comparisons is shown in Table 3. The high risk of both cardiovascular and bleeding events in patients with AF-NAFLD was consistent in both males and females, and no statistically significant differences were found when the sexes were directly compared.

**Table 3.** Sensitivity analyses after propensity score matching

	Composite cardiovascular outcome		Composite hemorrhagic outcome	
	Events n (%)	HR (95% CI)	Events n (%)	HR (95% CI)
AF NAFLD males n = 8010 No liver disease males n = 8010	2611 (32.6) 1836 (22.9)	1.50 (1.41-1.59)	576 (7.2) 367 (4.6)	1.60 (1.40-1.82)
AF NAFLD females n = 6943 No liver disease females n = 6943	2269 (32.7) 1639 (23.6)	1.46 (1.37-1.56)	535 (7.7) 384 (5.5)	1.43 (1.25-1.63)
AF NAFLD males n = 10 752 AF NAFLD females n = 10 752	1482 (13.8) 1472 (13.7)	1.02 (0.95-1.09)	810 (7.5) 914 (8.5)	0.89 (0.81-0.98)
AF NAFLD obesity n = 2699 No liver disease obesity n = 2699	918 (34.0) 693 (25.7)	1.38 (1.25-1.52)	194 (7.2) 141 (5.2)	1.39 (1.12-1.72)
AF NAFLD without obesity n = 9844 No liver disease without obesity n = 9844	3294 (33.5) 2219 (22.5)	1.59 (1.51-1.68)	710 (7.2) 518 (5.3)	1.43 (1.27-1.60)
AF NAFLD with obesity n = 4024 AF NAFLD without obesity n = 4024	1181 (29.3) 1237 (30.7)	0.83 (0.77-0.90)	366 (9.1) 253 (6.3)	1.23 (1.05-1.44)
NAFLD without cirrhosis n = 12 004	3909 (32.6)	1.49 (1.42-1.56)	882 (7.3)	1.53 (1.38-1.70)
No liver disease n = 12 004	2757 (23.0)		586 (4.9)	
NAFLD with cirrhosis n = 1558	625 (40.1)	2.30 (1.95-2.55)	172 (11.0)	2.39 (1.83-3.12)
No liver disease n = 1558	320 (20.5)		79 (5.1)	
NAFLD with cirrhosis n = 2326	930 (40.0)	1.40 (1.04-1.25)	270 (11.6)	1.52 (1.27-1.83)
NAFLD without cirrhosis n = 2326	839 (36.1)		194 (8.3)	
NAFLD without thrombocytopenia n = 11 244	3412 (30.4)	1.13 (1.07-1.18)	761 (6.8)	1.26 (1.13-1.40)
No liver disease n = 11 244	3001 (26.7)		586 (5.2)	
NAFLD with thrombocytopenia n = 1936	543 (28.0)	1.48 (1.35-1.63)	236 (12.2)	2.04 (1.64-2.55)
No liver disease n = 1936	340 (17.6)		118 (6.1)	
NAFLD with thrombocytopenia n = 3684	1923 (52.2)	1.53 (1.43-1.64)	490 (13.3)	1.70 (1.49-1.99)
NAFLD without thrombocytopenia n = 3684	1407 (38.2)		302 (8.2)	
NAFLD on warfarin n = 4131	1846 (44.7)	1.54 (1.44-1.66)	398 (9.6)	1.44 (1.23-1.67)
No liver disease on warfarin n = 4131	1316 (31.9)		287 (6.9)	
NAFLD on NOAC n = 15 619	4843 (31.0)	1.76 (1.68-1.84)	1122 (7.2)	1.80 (1.63-1.98)
No liver disease on NOAC n = 15 619	2897 (18.5)		627 (4.0)	
NAFLD on warfarin n = 8766	3860 (44.0)	1.28 (1.22-1.34)	874 (10.0)	1.22 (1.10-1.35)
NAFLD on NOAC n = 8766	3065 (35.0)		685 (7.8)	

Abbreviations: HR, hazard ratio; NAFLD, nonalcoholic fatty liver disease.

In the second sensitivity analysis, after PSM were selected for each group: (1) 2699 for the comparison between patients with AF-NAFLD and those with AF without liver disease, only obese; 9844 for the comparison between patients with AF-NAFLD and those with AF without liver disease only, nonobese; and 4024 for the comparison between obese patients with AF-NAFLD and nonobese patients with AF-NAFLD (Supplementary Tables S6-S8 (13)). The number of events reported after 1 year of follow-up in all the comparisons is shown in Table 3. Although the risk of cardiovascular and bleeding events in patients with AF-NAFLD was regardless of obesity, we found that patients with AF-NAFLD with obesity had a lower risk of composite cardiovascular outcomes compared to those nonobese (HR, 0.83; 95% CI, 0.77-0.90).

In the third sensitivity analysis, after PSM was selected for each group (1) 12 004 patients for the comparison between those with AF-NAFLD without cirrhosis and patients with AF without liver disease; (2) 1558 patients for the comparison between patients with AF-NAFLD with cirrhosis compared to patients with AF without liver disease; and (3) 2326 patients for the comparison between patients with AF-NAFLD with cirrhosis and patients with AF-NAFLD without cirrhosis (Supplementary Tables S9-S11 (13)). The number of events reported after 1-year follow-up in all the comparisons is shown in Table 3.

Patients with AF-NAFLD without and with cirrhosis showed a progressively increased risk of composite cardiovascular (HR, 1.49; 95% CI, 1.42-1.56; and HR, 2.23; 95% CI, 1.95-2.55, respectively) and hemorrhagic (HR, 1.53; 95% CI, 1.38-1.70; and 2.39; 95% CI, 1.83-3.18, respectively) outcomes compared to patients with AF without liver disease.

When directly compared, patients with AF-NAFLD with cirrhosis showed a significantly higher risk for both the composite cardiovascular (HR, 1.14; 95% CI, 1.04-1.25) and hemorrhagic (HR, 1.52; 95% CI, 1.27-1.83) outcomes compared to patients with AF-NAFLD without cirrhosis.

In the fourth sensitivity analysis after PSM, we selected for each group: (1) 11 244 patients for the comparison between patients with AF with NAFLD without thrombocytopenia vs those without liver diseases; (2) 1936 patients for the comparison between patients with AF NAFLD with thrombocytopenia vs those without liver diseases; and (3) 3684 patients for the comparison between patients with AF-NAFLD with thrombocytopenia vs those without (Supplementary Tables S12-S14 (13)). The number of events reported after 1 year of follow-up in all the comparisons is shown in Table 3. Both patients with AF-NAFLD without and with thrombocytopenia showed an increased risk of composite cardiovascular (HR, 1.13; 95% CI, 1.07-1.18; and HR, 1.48; 95% CI, 1.35-1.63, respectively) and hemorrhagic (HR, 1.26; 95% CI, 1.13-1.40; and HR, 2.04; 95% CI, 1.64-2.55, respectively) outcomes compared to patients with AF without liver disease. When directly compared, patients with AF-NAFLD with thrombocytopenia were associated with a 53% higher risk of the composite cardiovascular outcome and a 70% higher risk of the composite hemorrhagic outcome than patients with AF-NAFLD without thrombocytopenia (Table 3).

In the fifth sensitivity analysis after PSM, we selected for each group: (1) 4131 patients for the comparison between AF-NAFLD patients and AF patients without liver disease treated by warfarin; (2) 15 619 patients for the comparison between patients with AF-NAFLD and those with AF without liver disease treated with NOAC; and (3) 8766 patients for the comparison between patients with AF-NAFLD treated by warfarin compared to patients with AF-NAFLD treated with NOAC (Supplementary Tables S15-S17 (13)). The number of events reported after 1-year of follow-up in all the comparisons is shown in Table 3.

Both patients with AF-NAFLD treated with warfarin or NOAC were associated with a significantly increased risk of composite cardiovascular (HR, 1.54; 95% CI, 1.44-1.66; and HR, 1.76; 95% CI, 1.68-1.84, respectively) and hemorrhagic (HR, 1.44; 95% CI, 1.23-1.67; and HR, 1.80; 95% CI, 1.63-1.98, respectively) outcomes compared to patients with AF without liver disease. When directly compared, patients with AF-NAFLD on warfarin were associated with higher risks of the composite cardiovascular (HR, 1.28; 95% CI, 1.22-1.34) and hemorrhagic outcomes (HR, 1.22; 95% CI, 1.10-1.35) than patients with AF-NAFLD treated with NOAC (Table 3).

## Discussion

In this study, our principal findings are as follows: (1) patients with AF-NAFLD were younger but with a higher cardiovascular burden compared to patients with AF without liver disease; (2) AF-NAFLD was associated with a higher risk of all-cause death and cardiovascular and hemorrhagic events compared to patients with AF without liver disease; (3) the risk of adverse events was independent of sex, seems to be the highest in the nonobese, and progressively increased from noncirrhotic to cirrhotic and from nonthrombocytopenic to thrombocytopenic patients; and (4) the high risk of thrombosis and bleeding in patients with AF-NAFLD was independent of OAC type, but NOACs appeared to be safer and more effective compared to warfarin.

In this cohort, patients with AF-NAFLD were not only younger and at a higher cardiovascular risk, but were less likely to have been prescribed antihypertensive, hypolipidemic, and antiplatelet drugs. This suggests the presence of possible clinical concerns in initiating pharmaceutical treatments in patients with liver disease. Indeed, a cross-sectional study on 605 consecutive patients with NAFLD showed that about 50% of patients with indication to statin treatment, according to the European Society of Cardiology guidelines (14), do not receive any cholesterol-lowering medication (15). A retrospective study on 12 607 patients with NAFLD from the National Health and Nutrition Examination Survey found that 35.6% of patients with NAFLD had uncontrolled hypertension, whereas in 215 patients with AF with biopsy-proven nonalcoholic steatohepatitis from the Northwestern Medicine Enterprise Database Warehouse, only 62.5% received an appropriate OAC (16). Thus, the high prevalence of cardiovascular diseases and the lower use of cardiovascular medications could provide a first explanation for the high risk of cardiovascular events in AF-NAFLD before PSM. Indeed, before matching, we found a 2-fold increased risk for both the cardiovascular and hemorrhagic composite outcomes in patients with AF-NAFLD compared to those without liver disease, and this was consistent for each component of the composite outcomes. Hence, this could be related to the fact that NAFLD represents the expression of a broad metabolic dysfunction characterized by obesity, dyslipidemia, and insulin resistance, which could have mediated the effect on prognosis more than NAFLD itself (17-19). Nonetheless, when the 2 populations were propensity matched for each cardiovascular risk factor and pharmacological treatment, the results were consistent showing a possible independent role for the liver disease itself. Indeed, the risk of composite cardiovascular events was still 50% higher in AF-NAFLD compared to those with the same cardiovascular risk profile but without liver disease. This supports the findings from one prospective study on 898 patients with a least 1 component of the metabolic syndrome, where NAFLD was associated with a high cardiovascular risk (HR, 4.02; 95% CI, 1.21-13.38) (20); and from a meta-analysis on more than 5 800 000 middle-aged individuals, showing that patients with NAFLD have a 45% higher cardiovascular risk (HR, 1.45; 95% CI, 1.31-1.61) compared to those without liver disease (21).

Moreover, when analyzing the risks associated with each component of the cardiovascular composite outcome, our results were consistent with those reported in a meta-analysis of 20 different studies (22), showing that patients with NAFLD have a higher risk of myocardial infarction (OR, 1.66; 95% CI, 1.39-1.99) and ischemic stroke (OR, 1.41; 95% CI, 1.29-1.55). Similarly, a nationwide study performed on 472 212 Korean patients reported that NAFLD-related liver fibrosis was associated with a higher risk of venous thromboembolism (OR, 1.45; 95% CI, 1.30-1.62) (23).

Contrary to our findings, a prospective multicenter cohort study on 1735 patients with AF of whom 732 (42.2%) with NAFLD found no significant association between NAFLD and the incident risk of cardiovascular events (HR, 0.91; 95% CI, 0.64-1.29) (24). After adjustment for confounders, the risk of adverse events was associated with the metabolic syndrome components rather than with the NAFLD itself (25). Although the discrepancy with these studies could be partially explained by the different diagnostic criteria, the relatively small sample size, and the small number of events during the follow-up may be underpowered, necessitating further prospective studies in larger AF cohorts to better clarify this issue.

Furthermore, we observed that the risk of adverse events in patients with AF-NAFLD was irrespective of obesity, yet among patients with AF-NAFLD, the risk of composite cardiovascular outcomes was the highest in nonobese individuals. These findings align with recent evidence demonstrating the presence of an “obesity paradox” in patients with AF (26). The explanations behind this paradox are complex and involve several factors. Obesity status is often diagnosed using body mass index, which does not account for the ratio between lean and fat mass. The nonobese group may potentially include elderly, underweight, or cachectic patients who are at an increased risk of mortality. Additionally, obese patients, being afflicted by several cardiovascular risk factors and prescribed different medical drugs, could be more prone to undergo strict follow-up with early recognition of potentially harmful adverse events. However, although these hypotheses could in part explain our findings, further prospective studies are needed to better investigate the role of the obesity paradox in patients with AF-NAFLD (27).

What is recently emerging in patients with NAFLD is that the risk of cardiovascular events is directly related with liver fibrosis severity. In 898 patients prospectively followed for 41.4 months, for example, patients with NAFLD with mild or severe liver fibrosis had a progressively increased risk of cardiovascular events compared to patients without liver disease (20). This was confirmed by several other studies performed in different clinical contexts in which derived liver fibrosis indexes were associated with worse cardiovascular outcomes (28-31).

In our study, the risk of cardiovascular events in AF-NAFLD patients increased from noncirrhotic to cirrhotic patients and was the highest in the latter group. Liver cirrhosis is associated with an increased risk of both thrombosis and bleeding (32-35) because of the impairment of the liver metabolic functions and the hemodynamic changes present in cirrhotic patients (36). Moreover, cirrhotic patients may have thrombocytopenia that is characterized by a low number of hyperreactive platelets that could be associated with a high risk of both hemorrhagic and thrombotic events (37). However, the increased risk of adverse events even in AF-NAFLD without cirrhosis or thrombocytopenia suggests that similar pathological manifestations may be present since the earliest stages. Indeed, the pro-fibrotic response consequent to the uncontrolled hepatic fat accumulation induces a pro-thrombotic state even before extensive liver fibrosis is clinically manifest (38).

Finally, patients with AF-NAFLD in our cohort had a higher bleeding risk compared to patients with AF without liver disease and this was consistent for both ICH and gastrointestinal bleeding. Few previous studies have reported data about the bleeding risk in patients with NAFLD. A prospective study on 49 906 individuals from the Kailuan study, followed for a median of 6.79 years, found that the steatosis severity was directly correlated with the risk of ICH (HR, 2.33; 95% CI, 1.24-4.38) (39). Also, a retrospective study on 799 785 hospitalized patients derived from the National Inpatient Sample from the United States, showed that patients with NAFLD with nonvariceal gastrointestinal bleeding had an increased risk of death and hemorrhagic shock compared to those without liver disease (40). However, a prospective study on 1735 patients with AF found no association between NAFLD and the incident risk of major bleeding (HR, 0.85; 95% CI, 0.52-1.37) (24).

In our study, the increased risk of bleeding in AF-NAFLD was confirmed even in noncirrhotic patients, was independent of the OAC type, yet was the highest in those prescribed warfarin. Patients with NAFLD may have significant alterations in the activity of drug-metabolizing enzymes that affect the clearance of therapeutic agents (41). Indeed, CYPA2 is downregulated, whereas CYP2E1 and the efflux transporter MRP3 are upregulated, potentially inducing high variability in the OAC bioavailability in patients with NAFLD (11, 42). These alterations were more marked in those taking warfarin compared to NOAC. In a retrospective study on 430 patients with NAFLD and 38 887 patients without liver disease on warfarin, NAFLD was an independent predictor of lower average daily dose and shorter time in therapeutic range, particularly in those with obesity or diabetes (43). In a prospective study on 172 patients with AF-NAFLD and 428 patients with AF without liver disease, the therapeutic effect of warfarin was impaired in those with NAFLD (44).

International guidelines recommended that NOAC can be used in patients with liver cirrhosis with a Child-Pugh A or B, except for rivaroxaban, for which controversial data in patients with moderate liver impairment do exist (45, 46). However, these indications are based on studies performed with small cohorts and there are limited data about the NOACs in patients with liver disease. Indeed, these patients were excluded by most of the randomized clinical trials performed to assess NOAC safety and efficacy (47-50). A post hoc analysis of the ENGAGE AF-TIMI 48 (Effective Anticoagulation With Factor Xa Next Generation in Atrial Fibrillation-Thrombolysis In Myocardial Infarction Study 48) trial on 21 105 patients with AF, of whom 1083 (5.1%) with liver diseases, showed no difference for the risk of major bleeding and stroke in patients with AF with liver disease treated by edoxaban or warfarin (51). However, a metanalysis that included 3483 patients with AF with chronic liver disease showed that patients with AF treated with NOAC have a significant lower risk of major bleeding and a similar risk of stroke compared to those treated with warfarin (34).

Overall, the evidence supporting the heightened risk of adverse events associated with NAFLD underscores the necessity for a holistic care approach in these patients. Beyond stroke prevention with OACs, there is a pressing need for evidence supporting the beneficial effects of AF rhythm management in this patient subset. Multidisciplinary cardiovascular preventive strategies, encompassing optimization of comorbidities and lifestyle modifications, alongside the utilization of noninvasive tools for assessing and monitoring liver fibrosis progression, are essential for an integrated care approach to AF management. Indeed, adherence to the integrated Atrial fibrillation Better Care pathway has been associated with improved clinical outcomes in patients with AF (52, 53). Hence, further studies aimed at identifying the best clinical approaches in patients with AF-NAFLD are needed.

### Limitations

There are several limitations to acknowledge when interpreting the results of our study. First, the selection of patients with AF with liver disease was based on the current ICD-10-CM classification for NAFLD, which does not consider the recently introduced definition of metabolic dysfunction-associated steatotic liver disease (54). However, a previous study showed an almost complete overlap between the population historically identified as NAFLD and the population defined by the new metabolic dysfunction-associated steatotic liver disease diagnostic criteria (55). Second, administrative data may fail to identify a relatively significant proportion of patients with AF or NAFLD and thus may bias the selection of patients and their prognosis. Indeed, we cannot establish how the NAFLD diagnosis was made and if it was biopsy proven. Third, the competitive risk analysis showed a potential underestimation of the risk of most cardiovascular and hemorrhagic events considered in this analysis. This was more evident in patients with AF-NAFLD for stroke and myocardial infarction but could have also affected the risk of stroke in patients with AF without liver disease in the advanced phase of the follow-up. Fourth, we were not able to assess the fibrosis severity in either noncirrhotic or cirrhotic patients to detect the presence of differences in terms of risk of cardiovascular events or bleeding inside these 2 conditions. Fifth, the outcomes that occurred outside the network may have not been well captured and could have influenced the risks associated with the presence or absence of NAFLD. Sixth, the higher risk of adverse events observed in patients treated with warfarin compared to those on NOACs should be interpreted with caution. Currently, NOACs remain contraindicated in several conditions associated with a high thrombotic risk, such as advanced liver cirrhosis and end-stage renal disease, for which warfarin is still recommended. This could potentially bias the results. Further randomized clinical trials are necessary to elucidate the optimal antithrombotic strategies in patients with AF with NAFLD, particularly in those with advanced cirrhosis. Seventh, PSM has some limitations that should be kept in mind when interpreting the results. Indeed, although this statistical technique allows us to balance populations based on the prevalence of cardiovascular risk factors, it does not consider the severity of the disease. Moreover, although not statistically significant, patients with AF with NAFLD were still associated with a slightly higher absolute prevalence of some cardiovascular risk factors such as heart failure, ischemic heart disease, obesity, and chronic kidney disease after PSM, which may have influenced the risk of adverse events during follow-up. Finally, the study is limited by the inability to stratify the analysis according to ethnicity, or the presence of social disparities and thus gives just a partial landscape of this scenario.

## Conclusion

In patients with AF, NAFLD is associated with a higher 1-year risk of adverse events, with the risk of adverse events progressively increasing from noncirrhotic to cirrhotic and from nonthrombocytopenic to thrombocytopenic patients. NOACs were associated with a better outcomes and safety profile compared to warfarin as thromboprophylaxis.

## Data Availability

The data underlying this article are available on the TriNetX platform (https://www.trinetx.com).

## Abbreviations

AF: atrial fibrillation
HR: hazard ratio
ICD-10-CM: International Classification of Diseases, Tenth Revision, Clinical Modification
ICH: intracranial hemorrhage
NAFLD: nonalcoholic fatty liver disease
NOAC: non-vitamin K antagonist oral anticoagulant
OAC: oral anticoagulation
PSM: propensity score matching
